# Focused Ultrasound-Mediated Release of Bone Morphogenetic Protein 2 from Hydrogels for Bone Regeneration

**DOI:** 10.3390/gels11020120

**Published:** 2025-02-06

**Authors:** Tyus J. Yeingst, Angelica M. Helton, Ferdousi S. Rawnaque, Julien H. Arrizabalaga, Dino J. Ravnic, Julianna C. Simon, Daniel J. Hayes

**Affiliations:** 1 Department of Biomedical Engineering, The Pennsylvania State University, University Park, PA 16802, USA; tjy5139@psu.edu (T.J.Y.); amh8312@psu.edu (A.M.H.); jcs516@psu.edu (J.C.S.); 2 Graduate Program in Acoustics, The Pennsylvania State University, University Park, PA 16802, USA; fmr5186@psu.edu; 3 Huck Institutes of the Life Sciences, The Pennsylvania State University, University Park, PA 16802, USA; dur2@psu.edu; 4 Department of Surgery, Penn State Health Milton S. Hershey Medical Center, Hershey, PA 16802, USA; 5 Irvin S. Zubar Plastic Surgery Research Laboratory, Penn State College of Medicine, Hershey, PA 16802, USA; 6 Materials Research Institute, Millennium Science Complex, The Pennsylvania State University, University Park, PA 16802, USA

**Keywords:** Diels–Alder, click chemistry, controlled release, polyethylene glycol, hydrogel, bone defects, ultrasound

## Abstract

An ultrasound-responsive hydrogel system was developed that provides on-demand release when stimulated by focused ultrasound (fUS). Diels–Alder cycloadducts crosslinked polyethylene glycol (PEG) hydrogels and underwent a retrograde Diels–Alder reaction when exposed to fUS. Four-arm and eight-arm furan-based Diels–Alder hydrogel compositions were used to evaluate the link between the crosslinking density and the fUS-induced release and retention rates. PEG crosslinked with glutaraldehyde was also used as a non-Diels–Alder control hydrogel. By increasing the exposure time and the amplitude of fUS, the Diels–Alder-based hydrogels exhibited a correlative increase in the release of the entrapped BMP-2. Real-time B-mode imaging was used during fUS to visualize the on-demand degradation of the crosslinking matrix for the release of BMP-2. When monitored with a thermocouple, the increase in temperature observed was minimal in the area surrounding the sample during fUS stimulation, indicating fUS to be an external stimulus which could be used safely for spatiotemporally controlled release. PEG hydrogels were characterized using nuclear magnetic resonance, Fourier transform infrared spectroscopy, differential scanning calorimetry, thermogravimetric analysis, and compression testing. PEG degradation byproducts were evaluated for cytocompatibility in vitro. Overall, this study demonstrated that Diels–Alder-based PEG hydrogels can encapsulate BMP-2, undergo a retrograde reaction when externally stimulated with fUS, and release active BMP-2 to induce differentiation in human mesenchymal stem cells.

## 1. Introduction

The field of tissue engineering seeks to address many challenges which impact global health and the quality of life, including bone defects and diseases [1,2]. Bone defects are defined by gaps in bone tissue or structural abnormalities which impair the stability or function of the bone, often resulting from trauma, cancer, infection, surgical resection, congenital disorders, or degenerative conditions [1,2,3]. Among the key strategies for bone regeneration, the controlled delivery of bone morphogenetic protein-2 (BMP-2) or complementary growth factors has emerged as a therapeutic cornerstone due to the protein’s potent osteoinductive properties [4,5,6]. The efficacy of BMP-2 delivery systems remains limited by the need to balance spatiotemporal precision with sustained release to avoid exposing surrounding tissues to doses higher than normal physiological concentrations, which may lead to adverse effects such as ectopic bone formation, inflammation, heterotopic ossification, or tissue overgrowth [7,8,9]. Therefore, the development of biomaterials that enable the precise, on-demand, and localized release of BMP-2 while maintaining biocompatibility and stability represents an unmet need in the field.

Polyethylene glycol (PEG)-based hydrogels have gained widespread interest as scaffolds for tissue engineering and bone regeneration applications due to their tunable mechanical properties, hydrophilicity, and ability to encapsulate biomolecules [10,11,12,13]. Their chemical versatility permits the integration of functional groups that enable controlled drug release [14,15]. Recent advances have further expanded the potential of PEG hydrogels by incorporating stimuli-responsive mechanisms, which allow for the dynamic release of encapsulated factors in response to external stimuli [16,17,18,19]. Among these stimuli, focused ultrasound has emerged as a leading external trigger due to its noninvasive and nonionizing properties, deep tissue penetration, and spatiotemporal precision [19,20,21,22]. Ultrasound-responsive hydrogels, therefore, hold significant promise for applications where the precise control of therapeutic delivery is of high importance. Repeated applications of focused ultrasound could allow for a long-term step-like release profile meeting the clinical need for sustained release.

In parallel, the use of dynamic covalent chemistry, such as Diels–Alder (DA) reactions, in hydrogel crosslinking has revolutionized the design of injectable and responsive biomaterials [21,23,24,25]. DA reactions offer several unique advantages for biomedical applications, including reversibility under mild conditions, yielding the initial reactants, tunable reaction kinetics to ensure physiologically safe thermal responsivity, and synthesis methods compatible with bioactive molecules [26,27,28,29]. As a result of these characteristics, DA crosslinking enables the design of hydrogels with both structural integrity and dynamic responsivity [30,31,32]. By leveraging the synergy between ultrasound responsiveness and DA crosslinking, a new generation of PEG-based hydrogels can be designed to achieve the spatiotemporally controlled release of therapeutic agents like BMP-2.

Despite the potential of such systems, their application in bone regeneration has remained underexplored. Conventional BMP-2 delivery, which lacks spatiotemporal control, often results in overexposure and insufficient therapeutic localization [33]. Moreover, existing studies on ultrasound-triggered drug release have primarily focused on small molecules or general protein release, with limited emphasis on growth factors critical for regenerative processes [34,35,36]. As a result, the integration of ultrasound-responsive, DA-crosslinked PEG hydrogels containing BMP-2 introduces a unique platform for the customizable and localized delivery of proteins for bone regeneration and defect repair, addressing the challenges of conventional treatment methods.

**Figure 1:**
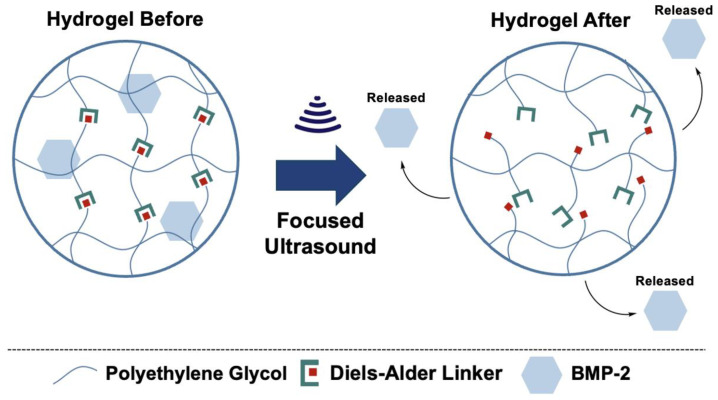
Conceptual design of ultrasound-responsive crosslinking network for spatiotemporally controlled release of BMP-2.

In this study, we present a novel ultrasound-responsive PEG hydrogel system crosslinked via Diels–Alder chemistry for the spatiotemporally controlled release of BMP-2. This hydrogel platform was engineered to encapsulate BMP-2 within a stable matrix that can be selectively disrupted by ultrasound (Figure 1), enabling on-demand release with high spatiotemporal precision. The Diels–Alder crosslinking mechanism provides structural tunability, which allows for the optimization of the hydrogel’s mechanical properties and degradation rates to mimic the physiological needs of bone regeneration [30,37,38,39]. In addition, the ultrasound-responsive component introduces an external trigger for the release of BMP-2, enhancing the spatiotemporal control over the dosing and distribution at the site of application. We hypothesized that this responsive hydrogel system would promote enhanced bone regeneration by addressing the limitations of conventional BMP-2 delivery approaches. To test this hypothesis, we conducted a comprehensive characterization of the physical and chemical properties. Following the characterization of the biomaterial, we evaluated the biocompatibility to ensure the efficacy of the material moving forward. Then, we evaluated the release kinetics in response to ultrasound stimulation, confirming the system’s ability to achieve localized release. Finally, the bone tissue regenerative potential of the system was assessed in an in vitro model, demonstrating enhanced bone formation.

This work highlights the potential of ultrasound-responsive DA-crosslinked PEG hydrogels as a versatile and effective platform for controlled BMP-2 release. The integration of the dynamic covalent DA chemistry, which responds to noninvasive external stimuli, provides a powerful tool to achieve spatiotemporal control in the release of therapeutic agents and subsequently paves the way for safer and more efficient regenerative therapies. Furthermore, the tunability of this platform provides the ability to deliver alternative or multiple growth factors in clinical applications beyond tissue engineering. This manuscript details the design, synthesis, characterization, biocompatibility, and efficacy of this innovative hydrogel system applied to bone regeneration through the differentiation of human-derived mesenchymal stem cells via released BMP-2. Through rigorous evaluation, we demonstrate that the combination of ultrasound-triggered release and DA crosslinking addresses key limitations in current BMP-2 therapies, through the delivery of physiologically relevant doses with spatiotemporal control.

## 2. Results and Discussion

### 2.1. Characterization and Mechanical Testing of PEG Diels–Alder Hydrogels

**Figure 2:**
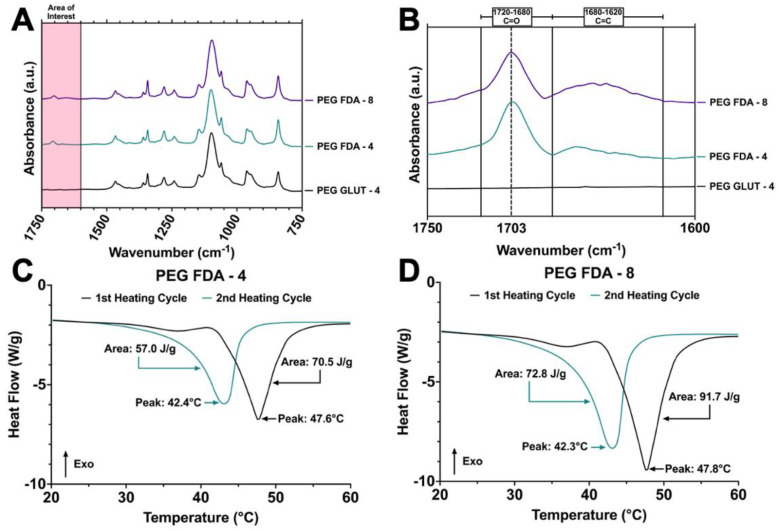
(**A**) Fourier transform infrared spectroscopy of final dried hydrogel matrices for PEG-FDA-4, PEG-FDA-8, and PEG-GLUT-4 from 750 cm^−^^1^ to 1750 cm^−^^1^. (**B**) Fourier transform infrared spectroscopy of final dried hydrogel matrices for PEG-FDA-4, PEG-FDA-8, and PEG-GLUT-4 in key area of interest between 1600 cm^−^^1^ and 1750 cm^−^^1^. (**C**) Differential scanning calorimetry of two sequential heating cycles from 20 °C to 60 °C with PEG-FDA-4 and (**D**) PEG-FDA-8.

**Figure A1:**
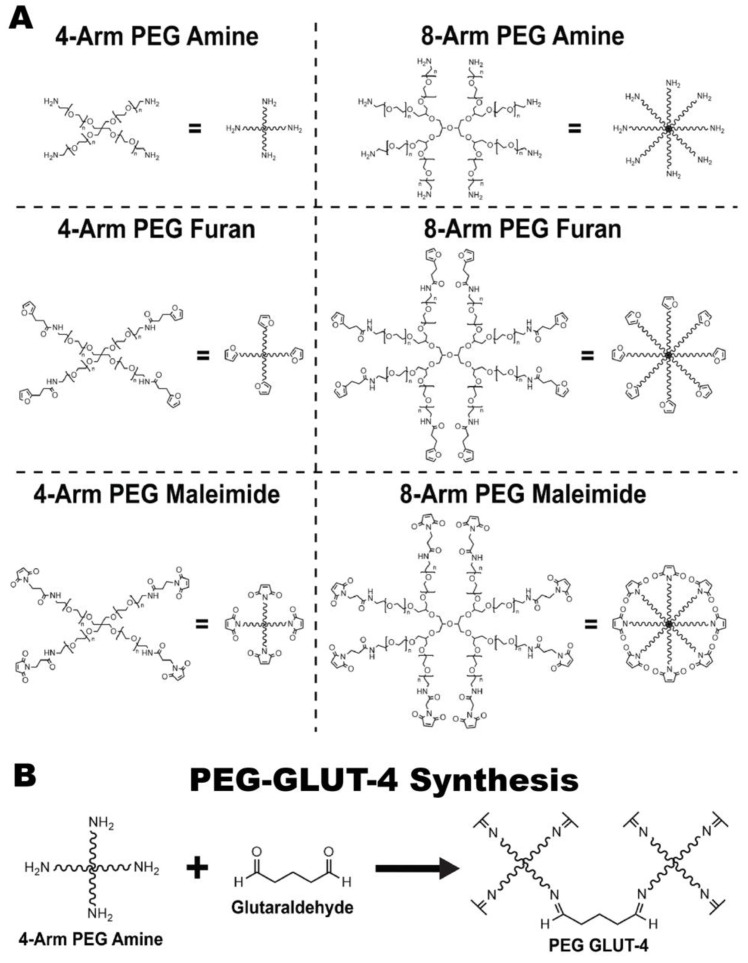
(**A**) Full chemdraws of synthesis intermediates. (**B**) Synthesis of PEG-GLUT-4.

**Figure A2:**
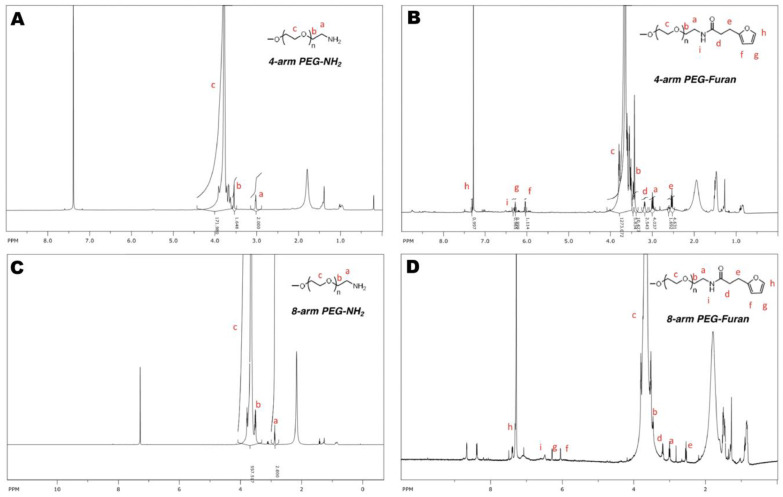
(**A**) The ^1^HNMR spectra of 4-arm PEG-amine, (**B**) 4-arm PEG-furan, (**C**) 8-arm PEG-amine, and (**D**) 8-arm PEG-furan.

**Figure A3:**
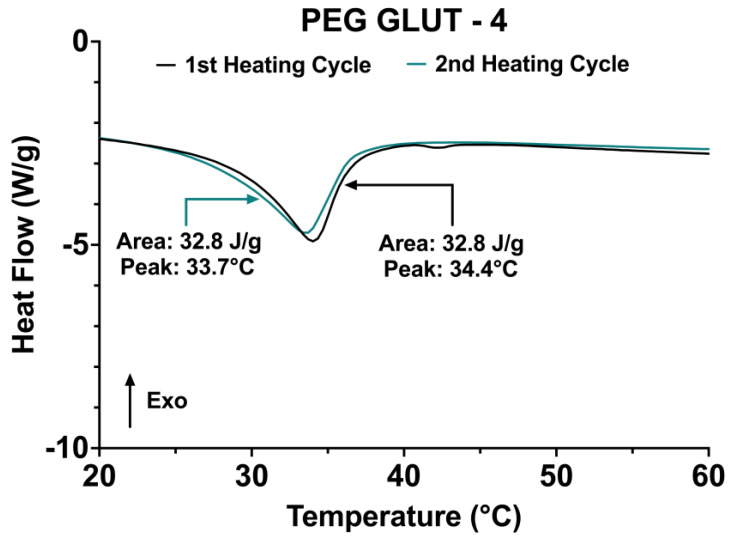
The DSC curves of the two increases in temperature from 20 °C to 60 °C from the 0 °C to 100 °C heating cycles of PEG-GLUT-4.

**Figure A4:**
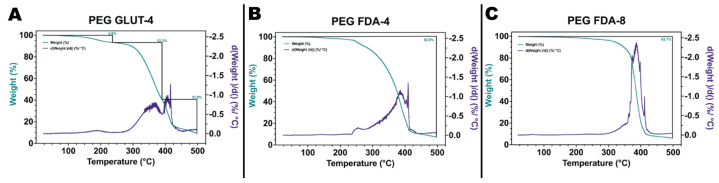
(**A**) Thermogravimetric analysis of PEG-GLUT-4, (**B**) PEG-FDA-4, (**C**) and PEG-FDA-8 from 20 °C to 500 °C.

**Figure A5:**
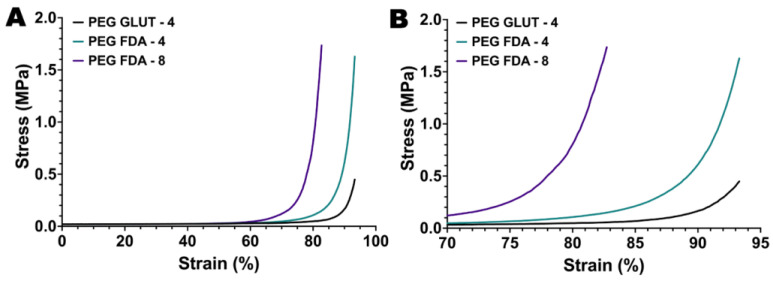
(**A**) Compression testing of PEG-GLUT-4, PEG-FDA-4, and PEG-FDA-8 with full strain percent range and (**B**) condensed range of 70% to 95% (*n* = 10).

The initial steps of PEG-FDA-4 hydrogel crosslinking began with a furan addition to four-arm PEG-amine (four-arm PEG-furan) and the addition of four-arm PEG-maleimide, resulting in PEG-FDA-4. The PEG-GLUT-4 hydrogel crosslinking began with a glutaraldehyde addition to four-arm PEG-amine, resulting in PEG-GLUT-4 (Appendix A Figure A1). The PEG-FDA-8 hydrogel crosslinking began with a furan addition to eight-arm PEG-amine (eight-arm PEG-furan as seen in Appendix A Figure A2) and the addition of eight-arm PEG-maleimide, resulting in PEG-FDA-8. The PEG-FDA-4, PEG-GLUT-4, and PEG-FDA-8 FTIR spectra are compared in Figure 2A,B. Characteristic absorption peaks can be seen at 1703 cm^−^^1^ between 1720 cm^−^^1^ and 1685 cm^−^^1^ for the C=O band and between 1680 cm^−^^1^ and 1620 cm^−^^1^ for the C=C band [40,41]. These FTIR bands represent differences resulting from the cycloaddition of Diels–Alder adducts when compared to the PEG-GLUT-4 control crosslinking method. Through FTIR, the confirmation of the final PEG Diels–Alder crosslinking was obtained prior to the reverse reaction temperature threshold assessment. PEG-FDA-4 and PEG-FDA-8 were analyzed using differential scanning calorimetry (Figure 2C,D). Two key peaks for the retro-DA reaction in PEG-FDA-4 were at 47.6 °C and 42.4 °C, with the peak at 47.6 °C taking place during the first heating cycle of 0 °C to 100 °C and the peak at 42.4 °C in the second heating cycle of 0 °C to 100 °C (Figure 2C). Two key peaks for the retro-DA reaction in PEG-FDA-8 were at 47.8 °C and 42.3 °C, with the peak at 47.8 °C taking place during the first heating cycle of 0 °C to 100 °C and the peak at 42.3 °C in the second heating cycle of 0 °C to 100 °C (Figure 2D). These peaks represent the temperature hydrogels needed to be in order to undergo a retrograde reaction the first and second time if applicable. The DA cycloadducts reverted to the diene and dienophile reactants from the original synthesis during the first cycle of heating before reforming, which is expected of DA hydrogel networks and has been previously described when two cycles of DSC heating are applied to DA constructs [42,43]. Two key peaks were measured for PEG-GLUT-4 as multiple melting points took place due to the reformation of hydrogel networks during cooling (Appendix A Figure A3). Thermogravimetric analysis was completed on PEG-FDA-4 and PEG-FDA-8, where both had a smooth decomposition curve with a weight percent loss of 92.8% or 93.7%, respectively, at 500 °C (Appendix A Figure A4B,C). Thermogravimetric analysis was also completed on PEG-GLUT-4 and showed a step-wise decomposition curve for the weight percent, resulting in a total decomposition of 90.4% at 500 °C (Appendix A Figure A4A). Compression testing was completed on PEG-FDA-4, PEG-FDA-8, and PEG-GLUT-4, where the final stresses were about 1.6, 1.75, and 0.4 MPa at around 93%, 82.5%, and 93.5% strain on average, respectively (Appendix A Figure A5).

### 2.2. Immersion Heating

**Figure A6:**
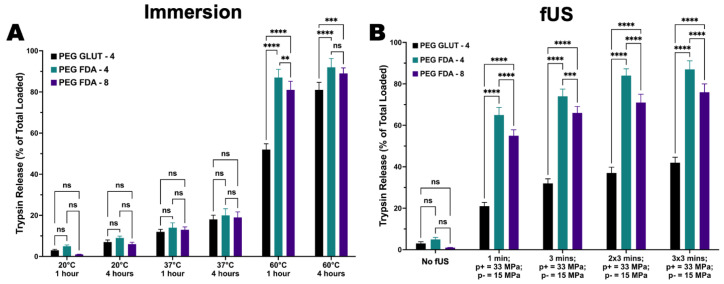
(**A**) Trypsin release as model payload using immersion bath at 20 °C, 37 °C, and 60 °C (*n* = 4, ns = no significance, ** = *p* < 0.01, *** = *p* < 0.001, **** = *p* < 0.0001). (**B**) Trypsin release as model payload using focused ultrasound targeting at peak positive pressure of 33 MPa and peak negative pressure of 15 MPa (*n* = 4, ns = no significance, *** = *p* < 0.001, **** = *p* < 0.0001).

**Figure A7:**
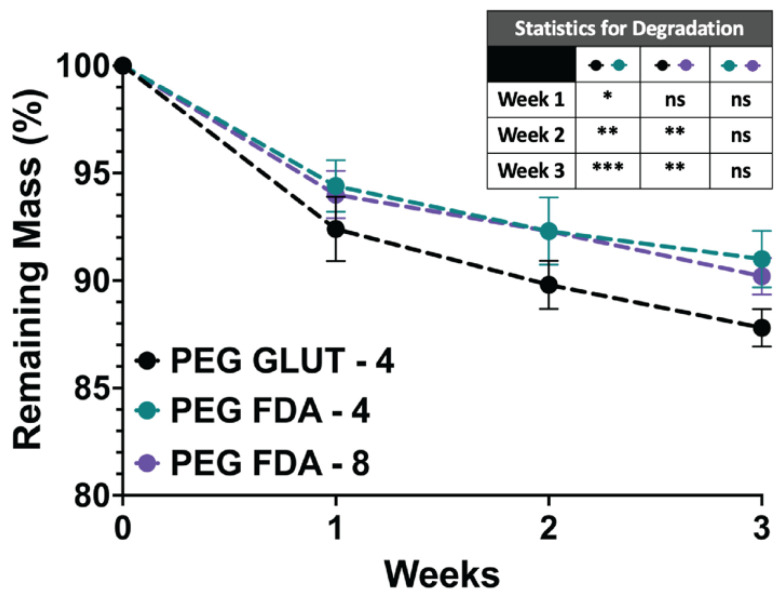
Mass degradation study of samples fully submerged in sealed microcentrifuge tubes containing 1 mL of DPBS at 37 °C for 1, 2, and 3 weeks (*n* = 4, ns = no significance, * = *p* < 0.05, ** = *p* < 0.01, *** = *p* < 0.001).

Immersion studies were performed on PEG-FDA-4, PEG-FDA-8, and PEG-GLUT-4 to measure the hydrogel degradation and release solely through thermal mechanisms. Measurements of the protein concentration were used to determine the amounts of trypsin (23.3 kDa) released via a retro-DA reaction when compared to a non-thermoresponsive crosslinking control, as visualized in Appendix A Figure A6A. A high trypsin release was correlated with higher thermal degradation in the samples. A higher protein release occurred in PEG-FDA-4 compared to PEG-FDA-8 under the same immersion heating conditions. This trend could be correlated with the crosslinking density, leading to a lower release as in previous studies [44,45]. Both PEG-FDA-4 and PEG-FDA-8 had a higher release than the non-thermoresponsive crosslinking control PEG-GLUT-4. This was expected because as the heat increases, the thermoresponsive crosslinked hydrogels will undergo a retrograde DA reaction, leading to the release of trypsin at higher rates. Trends for the long-term degradation of hydrogel samples at 37 °C are depicted in Appendix A Figure A7, where the mass loss after 1, 2, and 3 weeks was greater for PEG-GLUT-4 than for PEG-FDA-4 and PEG-FDA-8. The trend is as expected based on the differential scanning calorimetry data in Figure 2C,D, where PEG-FDA-4 and PEG-FDA-8 underwent a retrograde Diels–Alder reaction above 42 °C. PEG-GLUT-4, depicted in Appendix A Figure A3, showed the degradation of the crosslinking network below 37 °C.

### 2.3. Ultrasound-Induced Degradation and Model Release

**Figure A8:**
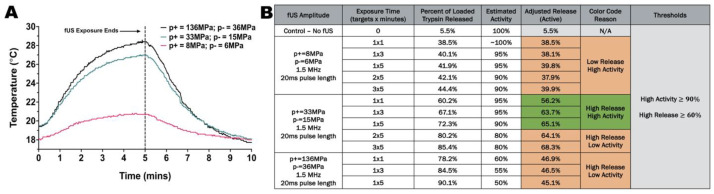
(**A**) Thermocouple temperature measurements 2 mm below sample at peak positive pressure of 8 MPa and peak negative pressure of 6 MPa, peak positive pressure of 33 MPa and peak negative pressure of 15 MPa, or peak positive pressure of 136 MPa and peak negative pressure of 36 MPa. (**B**) Evaluation of protein activity measured via BCA assay used to down-select ultrasound parameters based on high activity and high release rates (*n* = 5).

Focused ultrasound has been applied previously as an external stimulus that is both noninvasive and nonionizing for hydrogel degradation [46,47]. In this study, trypsin was entrapped in hydrogel matrices and the release was measured as an indicator of the focused ultrasound-induced degradation of the hydrogel. Appendix A Figure A6B indicates that fUS as an external stimulus drove the retro-DA reaction, causing trypsin release via hydrogel reorganization. These results suggest that fUS could be implemented in repeated treatments of hydrogels effectively to drive degradation; two and three treatments of fUS repeated at 3 min and 5 min intervals provided an expected increase in the trypsin release related directly to the quantity of the fUS exposure (Appendix A Figure A6B and Figure A8B). The “No fUS” groups were implemented to measure protein diffusion via the hydrolytic degradation of hydrogels under identical conditions with zero fUS exposure, indicating fUS to be an external stimulus that is effective in hydrogel reorganization and the release of trypsin. The release trends in this study compare well with the immersion heating conditions in Appendix A Figure A6A, suggesting that hydrogel degradation could be altered through crosslinking constructs by altering the crosslinking density and focused ultrasound parameters. Appendix A Figure A8A shows the change in the temperature of the area near hydrogel samples during fUS stimulation; these data were recorded with a thermocouple tip 2 mm beneath the sample at a peak positive pressure of 8 MPa and peak negative pressure of 6 MPa, peak positive pressure of 33 MPa and peak negative pressure of 15 MPa, or peak positive pressure of 136 MPa and peak negative pressure of 36 MPa. This temperature increase of about 7 °C would leave the tissue surrounding stimulation in vivo below 44 °C, demonstrating that fUS could be used as a safe and effective external stimulus for DA hydrogel degradation and release in vivo [48]. A secondary study was completed where lower acoustic pressures caused a lower protein release and smaller temperature increases (Appendix A Figure A8A,B). The results confirm that the hydrogel degradation and release rates directly correlated with the focused ultrasound energy and time targeted.

**Figure 3:**
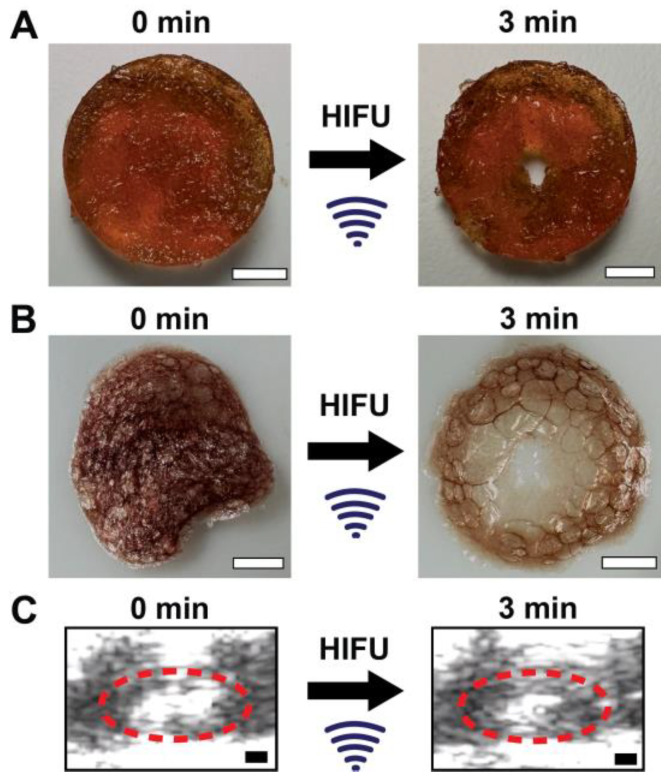
(**A**) An 8 mm PEG-FDA-4 hydrogel with no focused ultrasound exposure (**left**) and a hydrogel with 3 min of fUS exposure at 1.5 MHz with 20 ms repeated pulses at 1 Hz and *p*+ = 33 MPa and *p*- = 15 MPa (**right**) (scale bar = 2 mm). (**B**) The samples from A after being dried in a desiccator to visualize the crosslinking matrices (scale bar = 2 mm). (**C**) Real-time ultrasound imaging of PEG-FDA-4 before (**left**) and after 3 min of focused ultrasound (**right**), where the white space inside the red circle is the entirety of the PEG hydrogel (scale bar = 2 mm).

The focused ultrasound parameters of a peak positive pressure of 33 MPa and peak negative pressure of 15 MPa were used moving forward as per the previous protein activity study in Appendix A Figure A8B based on the protein release and protein activity following focused ultrasound exposure. Figure 3A shows PEG-FDA-4 and before and after 3 min of fUS exposure, with the B-mode real-time imaging of PEG-FDA-4 during focused ultrasound stimulation shown in Figure 3C. For the DA sample PEG-FDA-4 in Figure 3A, focused ultrasound reorganized the hydrogel fully, generating a cavity in the matrix with 3 min of ultrasound exposure. These two samples were then dried in a desiccator to better visualize the crosslinking matrix (Figure 3B). From the images in Figure 3B, it can be assumed that the energy from focused ultrasound did not only have an effect on the focal point where the hole was created but also the energy dispersed throughout the hydrogel. This can be seen in the thinning of the dried gel in Figure 3B.

### 2.4. Cytocompatibility

**Figure 4:**
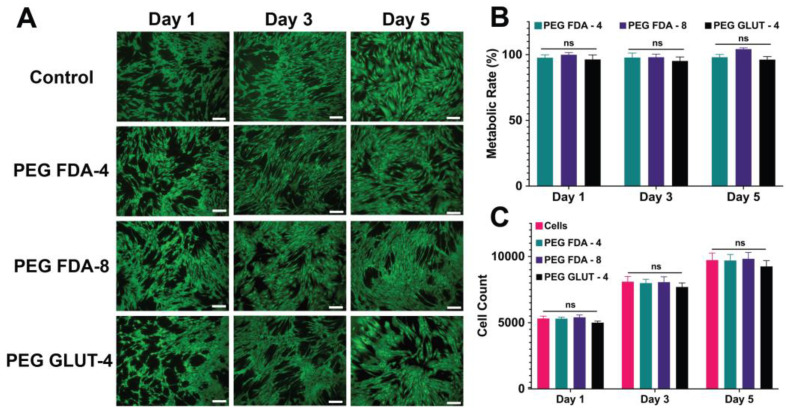
(**A**) Representative images of LIVE/DEAD staining for BMSCs at 1, 3, and 5 days of exposure to PEG hydrogel byproduct media. Scale bar = 200 μm. (**B**) The metabolic activity compared to BMSCs cultured with no exposure to PEG hydrogels (*n* = 5, ns = no significance). (**C**) The total cell count of BMSCs cultured with and without exposure to degraded PEG hydrogels (ns = no significance, *n* = 5).

The initial reactants for PEG-FDA-4 included four-arm PEG-furan and four-arm PEG-maleimide, while the initial reactants for PEG-FDA-8 included eight-arm PEG-furan and eight-arm PEG-maleimide. Cell media containing the respective focused ultrasound-induced PEG hydrogel degradation byproducts were used to assess the cytocompatibility of dienes and dienophiles in vitro. The metabolic rate, total cell count, and cell viability were evaluated (Figure 4). BMSCs were assessed in culture with a medium containing a 10% *w*/*v* ratio of degradation products (PEG-FDA-4, PEG-FDA-8, and PEG-GLUT-4) and then compared to BMSCs in culture with media not containing PEG hydrogel byproducts. DA PEG hydrogels did not show cytotoxic effects during exposure to BMSCs in vitro. When compared to a no-PEG hydrogel control group with only cells, no visible difference was seen in the LIVE/DEAD images of DA hydrogels (Figure 4A). Similarly, as shown in Figure 4B, there were no significant differences in the metabolic rate shown in the PEG-FDA-4 and PEG-FDA-8 groups when compared to the BMSC control (100%). There were also no significant differences shown in the cell count for PEG-FDA-4 and PEG-FDA-8 when compared to the BMSC-only control (Figure 4C). The control group PEG-GLUT-4 also exhibited no visible differences in any of the LIVE/DEAD images, as well as no significant difference in the metabolic rate or cell count (Figure 4). This was to be expected due to PEG’s high biocompatibility as described in the literature [12,49].

### 2.5. Retention and fUS-Induced Release of BMP-2

**Figure 5:**
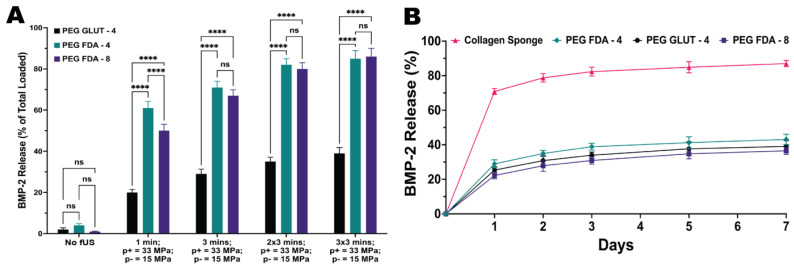
(**A**) Hydrogels loaded with BMP-2 underwent fUS exposure under the parameters of 1.5 MHz and a 20 ms pulse length with a peak positive pressure of 33 MPa and peak negative pressure of 15 MPa. Hydrogels were exposed to targeting once for 1 min and 1, 2, and 3 times for 3 min (*n* = 5, ns = no significance, **** = *p* < 0.0001). (**B**) Hydrogel matrices were compared to a collagen sponge through an immersion study over a 7-day period at 37 °C to measure the retention of BMP-2 (*n* = 5). Statistics for the retention study can be found in Appendix A.

**Figure A9:**
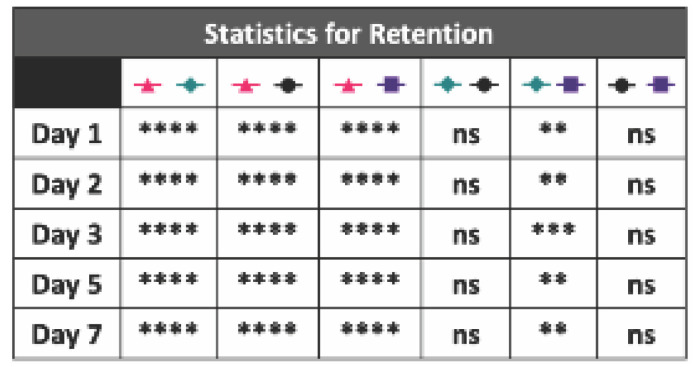
Statistics for the BMP-2 hydrogel and collagen sponge retention study (*n* = 5, ns = no significance, ** = *p* < 0.01, *** = *p* < 0.001, **** = *p* < 0.0001).

Hydrogels were loaded with BMP-2 as the final payload and the drug retention was compared to the current standard, a collagen eluting sponge. As depicted in Figure 5B and Appendix A Figure A9, all three PEG hydrogel constructs maintained a much higher BMP-2 retention than the current standard of the collagen sponge. This can be attributed to the higher crosslinking density of the hydrogels when compared to the large pore size of collagen sponges, also leading to higher spatiotemporal control over the release via external stimuli.

The hydrogels were again loaded with BMP-2 as the payload and underwent focused ultrasound stimulation to evaluate the spatiotemporal control over the release. Ultrasound parameters of 1.5 MHz and a 20 ms pulse length with a peak positive pressure of 33 MPa and peak negative pressure of 15 MPa were chosen according to the protein activity study in Appendix A Figure A6B. Figure 5A shows the BMP-2 release scales with both the time exposed to focused ultrasound and the number of exposures for each hydrogel sample. As expected, the results of the release studies for BMP-2 were highly similar to those of the model payload study using trypsin; therefore, the scaling was similar with the ultrasound parameters, also due to the similar size in kDa. We hypothesize that in order to obtain a prolonged step-like release over time, focused ultrasound could be used in small doses with multiple targets of the same hydrogel over the span of days to weeks between targets.

### 2.6. Differentiation of BMSCs from fUS-Induced Release of BMP-2

**Figure 6:**
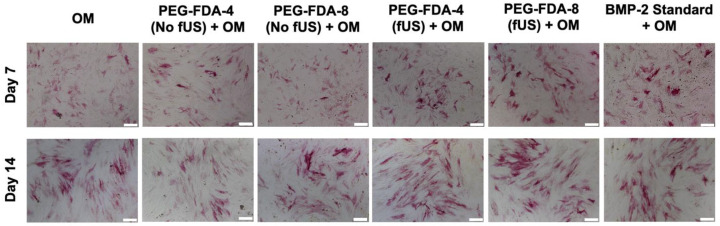
Alkaline phosphatase staining of BMSCs at days 7 and 14 after exposure to BMP-2, in comparison to standard BMP-2 stock solution and osteogenic medium controls. Statistics for well scans can be found in Appendix A Figure A10 (*n* = 5, scale bar = 200 μm).

**Figure 7:**
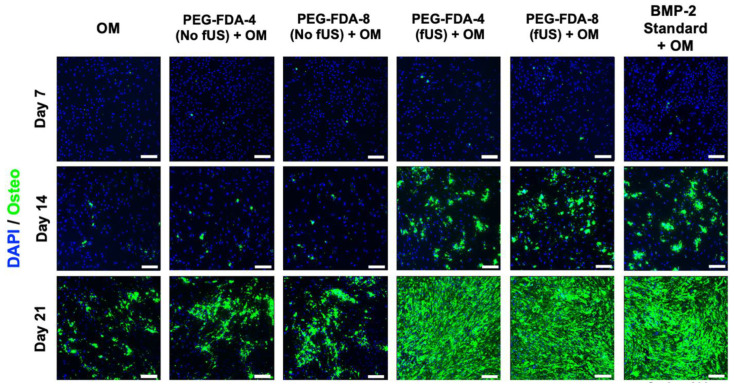
OsteoImage staining of BMSCs at days 7, 14, and 21 after exposure to BMP-2, in comparison to standard BMP-2 stock solution and osteogenic medium controls. Statistics for well scans can be found in Appendix A Figure A11 (*n* = 5, scale bar = 200 μm).

**Figure A10:**
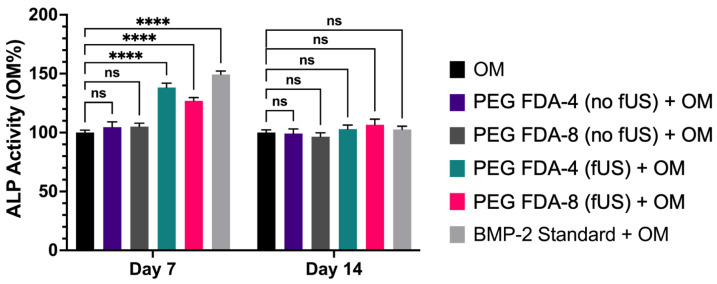
Results from 50 scans per well of the alkaline phosphatase stain measured on a Molecular Devices SpectraMax iD3 microplate reader (*n* = 5, ns = no significance, **** = *p* < 0.0001).

**Figure A11:**
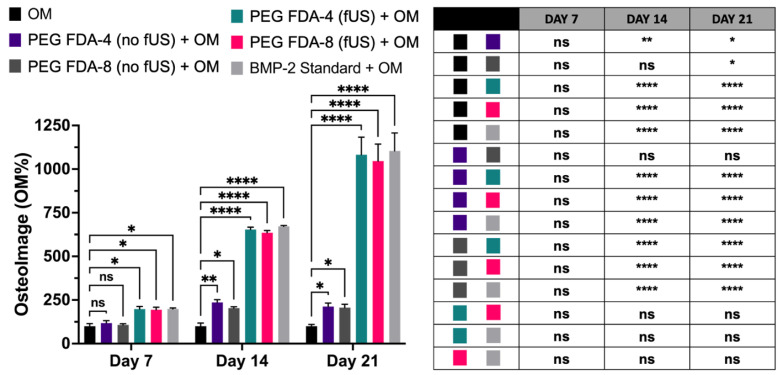
Results from 50 scans per well of the OsteoImage mineralization stain measured on a Molecular Devices SpectraMax iD3 microplate reader (*n* = 5, ns = no significance, * = *p* < 0.05, ** = *p* < 0.01, **** = *p* < 0.0001).

All six groups of BMSCs were cultured in an osteogenic medium (OM). Following the ultrasound-stimulated release from DA hydrogel groups, the BMP-2 concentration was standardized to 100 ng/mL and compared to a stock BMP-2 solution. Then, in a transwell model, the PEG-FDA-4 and PEG-FDA-8 hydrogels were submerged in media and allowed to elute BMP-2. Groups listed as “No fUS” were allowed to elute with no additional stimulation, while the fUS hydrogel groups contained the released 100 ng/mL injected into the transwell. BMSCs were stained for alkaline phosphatase at days 7 and 14 as a measure for early bone differentiation. The imaging of alkaline phosphatase staining is depicted in Figure 6, and the results from 50 scans per well of the alkaline phosphatase stain can be seen in Appendix A Figure A10. At day 7, only the fUS groups and the stock BMP-2 solution had significant increases in the alkaline phosphatase expression when compared to the OM control. At day 14, there were no significant differences in the alkaline phosphatase expression in any group when compared to the OM control group. BMSCs were stained for OsteoImage mineralization at days 7, 14, and 21, as a measure for late osteogenic differentiation. The imaging of the OsteoImage mineralization is depicted in Figure 7, and the results from 50 scans per well of the OsteoImage mineralization can be seen in Appendix A Figure A11. At day 7, only the fUS groups and the stock BMP-2 solution had significant increases in the OsteoImage mineralization when compared to the OM control (2-fold). At day 14, the fUS groups and the stock BMP-2 solution had significant increases in the OsteoImage mineralization when compared to the OM control (6-fold). At day 21, the fUS groups and the stock BMP-2 solution all had significant increases when compared to an OM control (10-fold). There were no significant differences between the fUS groups and the stock BMP-2 solution at any timepoint, indicating that there was no significant loss in BMP-2 activity following fUS exposure.

## 3. Conclusions

In this study, our group successfully crosslinked polyethylene glycol hydrogels via DA crosslinking. When our DA hydrogels were exposed to fUS, they underwent a retro-DA reaction causing hydrogel reorganization and the release of BMP-2. The release was shown to have tunability through the altered compositions and differing focused ultrasound parameters. Enhancing the cavitation and thermal bioeffects with fUS proved effective for hydrogel degradation and release, while enabling us to monitor the external stimuli effects in real time. It was also shown that increasing the time of exposure and fUS energy corresponded with an expected increase in the release among DA PEG hydrogel samples, which was much greater than the differences in the PEG-glutaraldehyde controls. The correlation with the BMP-2 release is suggestive of the on-demand spatiotemporal control afforded by using fUS for the external stimulus. Diels–Alder hydrogel degradation products did not show cytotoxicity during the in vitro evaluations. Additionally, the fUS-induced release of BMP-2 proved effective in both early and late bone differentiation when compared to both positive and negative controls. With the results from these studies, this hydrogel system could be developed effectively for in vivo evaluations and clinical research applications that have a need for or require the on-demand release of BMP-2. Future studies in vitro could include the analysis of differentiation rates dependent on the release dosage and time between doses in order to better understand how these parameters correlate with the BMSC differentiation rates. Future studies in vivo could include a long-term bone regeneration study with a comparison to the current standard methods of BMP-2 release such as collagen eluting sponges. The expected work for the future could include ultrasound-mediated drug release from hydrogels, with clinical applications in the fields of tissue engineering and cancer therapy. The technology created could be used in clinical applications for treating large bone defects caused by trauma, infection, tumor resection, or skeletal abnormalities.

## 4. Materials and Methods

### 4.1. Materials

We obtained 4-arm polyethylene glycol-amine (4-arm PEG-NH_2_, average MW of ~10,000), 4-arm polyethylene glycol-maleimide (4-arm PEG-Mal, average MW of ~10,000), 8-arm polyethylene glycol-amine (8-arm PEG-NH_2_, average MW of ~10,000), and 8-arm polyethylene glycol-maleimide (8-arm PEG-Mal, average MW of ~10,000) from Biopharma PEG Scientific (Watertown, MA, USA). *N*,*N*-diisopropylethylamine (DIPEA, ≥99%), FITC-BSA (bovine albumin), a glutaraldehyde solution (25% in H_2_O), Proteinase K (from Tritirachium album, ≥30 units/mg protein), and Dulbecco′s Phosphate-Buffered Saline (DPBS), chloroform-d (CDCl_3_, 99.8 atom % D), L-ascorbic acid 2-phosphate, β-glycerol phosphate, an alkaline phosphatase detection kit, human mesenchymal stem cells from bone marrow (BMSC, SCC034), and dexamethasone were acquired from Millipore Sigma (St. Louis, MO, USA). *N*,*N*-dimethylformamide (DMF, ≥99%), diethyl ether (anhydrous, ≥99.7%), 3,2-furyl propionic acid (≥97%), a 35 mm Nunc Petri dish, a Quant-iT PicoGreen dsDNA assay kit, a LIVE/DEAD Viability/Cytotoxicity kit, MEM α with nucleosides, L-glutamine, trypsin–EDTA, a bicinchoninic acid (BCA) assay, and penicillin/streptomycin (5000 U/mL) were acquired from Thermo Fisher Scientific (Waltham, MA, USA). Fetal bovine serum (FBS), 24-well tissue culture plates, 24-well transwell tissue culture plates (6.5 mm diameter inserts, 8.0 μm pore size), and 0.20 μm filters for media were acquired from Corning (Corning, NY, USA). HATU (≥98%) was acquired from Tokyo Chemical Industry (Tokyo, Japan). A CellTiter-Blue cell viability assay was purchased from Promega (Madison, WI, USA). Tegaderm was purchased from 3 M Health Care (St. Paul, MN, USA). A BMP-2 Quantikine ELISA Kit was purchased from bio-techne (Minneapolis, MN, USA). An Osteoimage mineralization assay was acquired from Lonza Bioscience (Basel, Switzerland). Human Recombinant BMP-2 was acquired from STEMCELL Technologies (Vancouver, BC, Canada).

### 4.2. Synthesis

**Figure 8:**
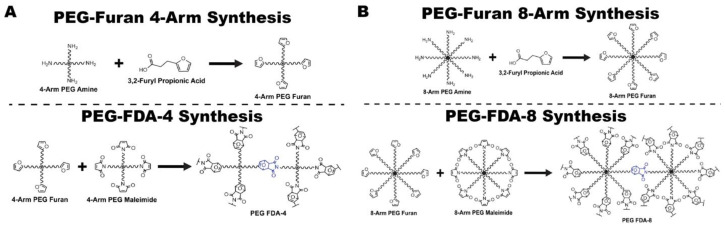
(**A**) Synthesis of 4-arm PEG-furan and PEG-FDA-4 via furan–maleimide-based Diels–Alder. (**B**) Synthesis of 8-arm PEG-furan and PEG-FDA-8 via furan–maleimide-based Diels–Alder.

Two furan-based DA (FDA) PEG hydrogels were synthesized for this study: PEG-FDA-4, which was created through the crosslinking of 4-arm PEG-furan with 4-arm PEG-maleimide, and PEG-FDA-8, which was the product of the crosslinking of 8-arm PEG-furan with 8-arm PEG-maleimide. We synthesized 4-arm PEG-furan and 8-arm PEG-furan from 4-arm PEG-amine and 8-arm PEG-amine, respectively. The control, PEG-glutaraldehyde, was created by crosslinking PEG-amine with glutaraldehyde. The entire multipart synthesis of 4-arm PEG-FDA and 8-arm PEG-FDA is described in Figure 8. The full Chemdraw structures of all intermediates and the synthesis of PEG-glutaraldehyde are available in Appendix A Figure A1.

#### 4.2.1. Preparation of 4-Arm PEG-Furan

The first solution contained 400 mg of 4-arm PEG-amine (10 kDa) dissolved in 12 mL of DMF, and it was stirred at room temperature for 15 min in a 50 mL round bottom glass flask. The second solution, which contained 89.6 mg of 3,2-furyl propionic acid and 365.2 mg of HATU, was dissolved in 8 mL of DMF and stirred at room temperature. A volume of 334.4 μL of DIPEA was added to the second stirred solution; this was stirred for 10 min in a glass vial. Finally, the second solution was added dropwise to the first solution and stirred at room temperature overnight. It was then precipitated in excess diethyl ether and washed 3 times before being dried under nitrogen overnight. This drying process resulted in the final product, 4-arm PEG-furan, as depicted in Figure 8A [50].

#### 4.2.2. Preparation of PEG-FDA-4

The first solution of 75 mg of 4-arm PEG-furan was dissolved in 1.5 mL of water with the selected model therapeutic or BMP-2. The second solution of 75 mg of 4-arm PEG-maleimide was dissolved in 1.5 mL of water. The second solution was added to the first solution and vortexed to ensure an even payload distribution. This 3 mL solution was then poured into a 35 mm diameter Petri dish. It was placed in an incubator overnight to allow for the crosslinking of the PEG-FDA-4 hydrogel, as depicted in Figure 8A.

#### 4.2.3. Preparation of 8-Arm PEG-Furan

The first solution of 400 mg of 8-arm PEG-amine (10 kDa) was dissolved in 15 mL of DMF and stirred at room temperature for 15 min in a 50 mL round bottom glass flask. The second solution of 179.2 mg of 3,2-furyl propionic acid and 730.4 mg of HATU was dissolved in 15 mL of DMF and stirred at room temperature. A volume of 668.8 μL of DIPEA was added to the second stirred solution and stirred for 10 min at room temperature in a glass vial. Finally, the second solution was added dropwise to the first solution and stirred at room temperature overnight. It was then precipitated in excess diethyl ether and washed 3 times before being dried under nitrogen overnight. This drying process resulted in the final product, 8-arm PEG-furan, as depicted in Figure 8B [50].

#### 4.2.4. Preparation of PEG-FDA-8

The first solution of 75 mg of 8-arm PEG-furan was dissolved in 1.5 mL of water with the selected model therapeutic or BMP-2. The second solution of 75 mg of 8-arm PEG-maleimide was dissolved in 1.5 mL of water. The second solution was added to the first solution and vortexed to ensure an even payload distribution. This 3 mL solution was then poured into a 35 mm diameter Petri dish. It was placed in an incubator overnight to allow for the crosslinking of the PEG-FDA-8 hydrogel, as depicted in Figure 8B.

#### 4.2.5. Preparation of PEG-GLUT-4

A total of 150 mg of the respective 4-arm and 8-arm PEG-amine was dissolved in 2.985 mL of water with the selected model therapeutic or BMP-2. A volume of 15 μL of a glutaraldehyde solution was then added and vortexed for an even distribution of payloads. The final solution was then poured into a 35 mm diameter Petri dish and placed in an incubator overnight to allow for crosslinking. The final product was PEG-GLUT-4 and is depicted in Appendix A Figure A1B.

#### 4.2.6. Preparation of Osteogenic Medium

First, 100 mL of FBS was added to 500 mL of MEM α with nucleosides. Then, 6 mL L-glutamine (200 mM in 0.85% NaCl; final concentration of 2–4 mM) and 6 mL penicillin/streptomycin (final concentration of 100 units/mL and 100 μg/mL streptomycin) were added to the media. Dexamethasone was added with a final concentration of 10 nM, β-glycerol phosphate was added with a final concentration of 20 mM, and L-ascorbic acid 2-phosphate was added with a final concentration of 50 μM. The medium was then filtered with a 0.20 μm filter and stored at 4 °C.

### 4.3. Experimental Approach

#### 4.3.1. Characterization and Mechanical Testing of Hydrogel Matrices

The differential scanning calorimetry analysis of the hydrogels was completed on a DSC Q2000 from TA (New Castle, DE, USA). The thermal properties of the materials were recorded at heating and cooling rates of 20 °C/min, with a temperature range of 0 °C to 100 °C; nitrogen was used for the purging gas. The dried hydrogels started at 0 °C and were heated to 100 °C, then cooled to 0 °C, and heated a second time to 100 °C. A TA Instruments TGA 5500 (New Castle, DE, USA) was used for the thermogravimetric analysis (TGA) of the hydrogels. The thermal properties were recorded while heating the hydrogels from 20 °C to 500 °C (Appendix A Figure A4). Fourier transform infrared (FTIR) spectra were measured using a Bruker V70 (Billerica, MA, USA) across the range of 4000 cm^−^^1^ to 600 cm^−^^1^. The ^1^H NMR spectra for intermediate compounds were measured with a Bruker Avance III HD 500 (Billerica, MA, USA) (CDCl3, 295 K, 500 MHz) and processed using Spinworks (University of Manitoba, Canada) as depicted in Appendix A Figure A2. Hydrogel samples were cut with a biopsy punch with a diameter of 8 mm and depth of 5 mm. Tensile testing was then completed at 37 °C using an MTS Criterion frame with MTS Fundamental Compression platens, which had a 50 kN capacity (Appendix A Figure A5).

#### 4.3.2. Real-Time Temperature During Focused Ultrasound Exposure

To measure the temperature of hydrogels during fUS exposure, the hydrogels were cast in a custom no-bottom well plate that was then sealed on both sides with Tegaderm. We used a thermocouple to measure the change in temperature of the area surrounding the hydrogel by placing it 2 mm below the hydrogel in water. A volume of 5 mm of water was injected above the sample to mimic the release studies. Sealed wells then underwent targeting via fUS. The temperature was measured at 1 measure/second with a Digi-Sense data logging thermocouple thermometer.

#### 4.3.3. Immersion Heating

Hydrogel samples containing trypsin (23.3 kDa) as a model payload were used to mimic BMP-2 (23.3–26 kDa) [51,52]. The hydrogels were cut using an 8 mm diameter biopsy punch (disposable). The hydrogels were then submerged in microcentrifuge tubes with 1 mL of DPBS above them. The tubes were sealed and then heated for 1 or 4 h at 20 °C, 37 °C, or 60 °C. The 20 °C tubes were kept in a room-temperature water bath, the 37 °C tubes were put into a heated water bath, and the 60 °C tubes were put into a heated oil bath. All microcentrifuge tubes were centrifuged at 1000× *g* for 10 min. The supernatant containing trypsin was then removed to assess the release from the crosslinked hydrogel matrices. This solution was separated into four aliquots of 200 μL. The concentration of trypsin released in each of the aliquots was assessed via a BCA assay used as per the manufacturer’s protocol and measured in a cuvette using a Thermo Scientific (Waltham, MA, USA) NanoDrop OneC set to 562 nm.

#### 4.3.4. Degradation Study

The hydrogels were cut with an 8 mm diameter biopsy punch. The hydrogels were then fully submerged and sealed within microcentrifuge tubes with 1 mL of DPBS above them. The tubes were heated to 37 °C for 1, 2, and 3 weeks in an incubator. Hydrogels were removed at 7, 14, and 21 days and dried in a Scienceware Lab Companion round vacuum desiccator and weighed.

#### 4.3.5. fUS-Induced Model Payload Release and Protein Activity Following fUS

In order to evaluate the protein release and activity following focused ultrasound, hydrogels were loaded with trypsin (23.3 kDa) as a model payload. All experiments were conducted in deionized water, degassed to achieve less than 20% dissolved oxygen. The hydrogels were targeted one, two, and three times for one, three, and five minutes. Focused ultrasound using a single-element focused ultrasound transducer with f# = 0.7 (H-234, Sonic Concepts, Bothell, WA, USA) at 1.5 MHz and a 20 ms pulse length with a pulse repetition frequency of 1 Hz was used at three settings: a peak positive pressure of 8 MPa and peak negative pressure of 6 MPa, a peak positive pressure of 33 MPa and peak negative pressure of 15 MPa, or a peak positive pressure of 136 MPa and peak negative pressure of 36 MPa. A waveform generator (Keysight, 33600A series, Colorado Springs, CO, USA) and RF amplifier (ENIA500, Rochester, NY, USA) were used to generate pulses. The released trypsin from each group was measured with a BCA assay used as per the manufacturer’s protocol and measured in a cuvette using a Thermo Scientific NanoDrop OneC set to 562 nm. The released trypsin was then compared to a stock trypsin solution to match the concentration, and it was evaluated for its activity via the lifting efficiency of the protein cleavage in cells.

#### 4.3.6. Imaging of fUS-Induced Degradation

Hydrogel samples were cut with a biopsy punch with a diameter of 8 mm. Samples were put in a custom no-bottom well plate that was sealed on both sides with Tegaderm, and 5 mm of water was placed on top of the sample to mimic the release studies. The samples were then targeted with fUS at 1.5 MHz and a 20 ms pulse length with a peak positive pressure of 33 MPa and peak negative pressure of 15 MPa for 3 min. During the targeting, real-time B-mode imaging (Verasonics, Kirkland, WA, USA) was used to visualize the degradation in real time. Following focused ultrasound exposure, the samples were compared to hydrogels without focused ultrasound exposure. Both samples were then dried in a Scienceware Lab (SP Bel-Art, Warminster, PA) Companion round vacuum desiccator to visualize the hydrogel crosslinking matrix pre- and post-focused ultrasound stimulation.

#### 4.3.7. Cell Viability and Proliferation Assays

BMSCs were cultured in an incubator at 37 °C with 5% CO_2_. The base cell medium was MEM α with nucleosides and ten percent injected fetal bovine serum and one percent injected penicillin/streptomycin. The PEG hydrogel byproduct media were obtained by degrading PEG-FDA-4, PEG-FDA-8, and PEG-GLUT-4 using focused ultrasound. The supernatant from the fUS exposure was removed from each group and dried under nitrogen. The dried fUS-induced degradation byproducts were then weighed and supplemented with MEM α until the media for the assays contained a 10% *w*/*v* ratio of the PEG byproducts for the respective crosslinking matrix; the individual media were then changed on the third day of each assay. Assays were then performed with initial seeding densities of 5000 cells per well in 24 wells and evaluated at days 1, 3, and 5. The metabolic activity of BMSCs was then evaluated using a CellTiter-Blue assay carried out as per the manufacturer’s protocol. The DNA count was evaluated using a PicoGreen dsDNA assay, and a LIVE/DEAD Viability/Cytotoxicity kit was used to visually evaluate the cell viability as previously described [21,53,54,55].

#### 4.3.8. Hydrogel Retention and fUS-Induced BMP-2 Release

In order to evaluate the hydrogel retention, hydrogels and a collagen sponge were loaded with BMP-2. The hydrogels were then submerged in DPBS and sealed in microcentrifuge tubes at 37 °C for 1, 2, 3, 5, and 7 days. At each timepoint, the supernatant was removed and the BMP-2 values were measured using a BMP-2 ELISA kit, which was used as per the manufacturer’s protocol at 450 nm using a Molecular Devices (San Jose, CA, USA) SpectraMax iD3 microplate reader.

In order to evaluate the hydrogel release following focused ultrasound, the hydrogels were loaded with BMP-2. The hydrogels were targeted once for one minute and one, two, and three times for three minutes. The fUS targeting parameters were 1.5 MHz and a 20 ms pulse length with a peak positive pressure of 33 MPa and peak negative pressure of 15 MPa. The released BMP-2 from each group was measured using a BMP-2 ELISA kit as per the manufacturer’s protocol at 450 nm using a Molecular Devices (San Jose, CA, USA) SpectraMax iD3 microplate reader.

#### 4.3.9. Alkaline Phosphatase Activity and Mineralization

BMSCs were cultured in an incubator at 37 °C with 5% CO_2_. Assays were then performed in 24-well transwell plates seeded at 5000 cells per well and measured at days 7 and 14 for the alkaline phosphatase (ALP) activity and 7, 14, and 21 days for the OsteoImage mineralization. All 6 groups of BMSCs were cultured in an osteogenic medium, or OM. Following the ultrasound-stimulated release from hydrogel groups, the BMP-2 concentration was standardized to 100 ng/mL and compared to a stock solution. Then, in a transwell model, the PEG-FDA-4 and PEG-FDA-8 hydrogels were submerged in media and allowed to elute BMP-2 over a 2- or 3-week period dependent on the assay length. Groups listed as “No fUS” were allowed to elute with no additional stimulation, while the fUS hydrogel groups contained the released 100 ng/mL injected into the transwell.

An alkaline phosphatase detection kit was then used at days 7 and 14 as per the manufacturer’s protocol as a widely accepted stain for early bone differentiation. Following staining, BMSCs were imaged using an Olympus Fluoview 1000 confocal microscope, and then the absorbance was measured at 405 nm using a Molecular Devices SpectraMax iD3 microplate reader with 50 scans per well.

An OsteoImage mineralization kit was used at days 7, 14, and 21 as per the manufacturer’s protocol as an indicator for late bone differentiation. Following staining, BMSCs were imaged using an Olympus Fluoview 1000 confocal microscope. Then, the fluorescent intensity was measured with an excitation and emission wavelength of 480/520 nm using a Molecular Devices SpectraMax iD3 microplate reader with 50 scans per well.

#### 4.3.10. Statistical Analysis for Studies

GraphPad Prism 10 was the statistical analysis software used. The statistical significance and sample size are located within each figure description. The results are shown with the mean ± the standard deviation. An ANOVA was used within the GraphPad Prism software to compare the groups.

## Data Availability

The raw data supporting the conclusions of this article will be made available by the authors on request.
